# Decreased HLA-DQ expression on peripheral blood cells in children with varying number of beta cell autoantibodies

**DOI:** 10.1016/j.jtauto.2020.100052

**Published:** 2020-04-09

**Authors:** Agnes Andersson Svärd, Marlena Maziarz, Anita Ramelius, Markus Lundgren, Åke Lernmark, Helena Elding Larsson, the DiPiS study group, C. Andersson, R. Bennet, I. Jönsson, M. Ask, J. Bremer, C. Brundin, C. Cilio, C. Hansson, G. Hansson, S. Ivarsson, B. Jonsdottir, B. Lindberg, B. Lernmark, J. Melin, A. Carlsson, E. Cedervall, B. Jönsson, K. Larsson, J. Neiderud

**Affiliations:** b Department of Clinical Sciences Malmö, Lund University, Sweden; c Department of Clinical Sciences Lund, Lund University, Sweden; d Department of Paediatrics, Ängelholm Hospital, Sweden; e Department of Paediatrics, Ystad Hospital, Sweden; f Department of Paediatrics, Kristianstad Central Hospital, Sweden; g Department of Paediatrics, Helsingborg Hospital, Sweden; a Department of Clinical Sciences, Lund University/CRC, Skåne University Hospital, 205 02, Malmö, Sweden

**Keywords:** Autoimmunity, Type 1 diabetes, Autoantibodies, Human Leukocyte antigen, Cell surface imunofluorescence

## Abstract

The risk for type 1 diabetes is strongly associated with HLA-DQ and the appearance of beta cell autoantibodies against either insulin, glutamate decarboxylase (GAD65), insulinoma-associated protein-2 (IA-2), or zinc transporter 8 (ZnT8). Prolonged exposure to autoantibodies may be related to T cell exhaustion known to occur in chronic infections or autoimmune disorders. It was hypothesized that autoantibody exposure may affect HLA-DQ expression on peripheral blood cells and thereby contribute to T cell exhaustion thought to be associated with the pathogenesis of type 1 diabetes. The aim of this study was to determine whether autoantibody exposure as an expression of autoimmunity burden was related to peripheral blood cell HLA-DQ cell surface expression in either 1) a cross-sectional analysis or 2) cumulative as area under the trajectory of autoantibodies during long term follow-up in the Diabetes Prediction in Skåne (DiPiS) study. Children (n = 67), aged 10–15 years were analyzed for complete blood count, HLA-DQ cell surface median fluorescence intensity (MFI), autoantibody frequency, and HLA genotypes by Next Generation Sequencing. Decreased HLA-DQ cell surface MFI with an increasing number of autoantibodies was observed in CD16^+^, CD14^+^CD16^−^, CD4^+^ and CD8^+^ cells but not in CD19^+^ cells and neutrophils. HLA-DQ cell surface MFI was associated with HLA-DQ2/8 in CD4^+^ T cells, marginally in CD14^+^CD16^−^ monocytes and CD8^+^ T cells. These associations appeared to be related to autoimmunity burden. The results suggest that HLA-DQ cell surface expression was related to HLA and autoimmunity burden.

## Highlights

• PBMC HLA-DQ surface expression in beta cell autoimmunity is poorly understood.
• Children, 10–15 years of age without or with beta cell autoantibodies were analyzed.
• HLA-DQ cell surface expression decreased with increasing number of autoantibodies.
• HLA-DQ cell surface expression was related to HLA and autoimmunity burden.

## 1 Introduction

Autoimmune (type 1) diabetes is strongly associated with HLA-DR-DQ; autoantibodies against beta cell autoantigens are robust biomarkers for beta cell autoimmunity and are strongly associated with an increased risk of progression to type 1 diabetes [[1], [2], [3], [4]]. Recent studies indicate that an association between the appearance of the first autoantibody and HLA is primary to the association between HLA and the clinically recognized disease [2,[5], [6], [7]]. Children who develop type 1 diabetes have one or more autoantibodies at diagnosis. However, the first appearing autoantibody precedes the clinical onset by months to years. Insulin autoantibodies (IAA) most often appears during the first two years of life, perhaps after a prolonged enterovirus infection [7,8], and is strongly associated with DR4-DQ8 [2]. The peak incidence of IAA decreases to very low levels by 8–10 years of age [3,4]. In contrast, glutamate decarboxylase autoantibodies (GADA) appears primarily in DR3-DQ2 children, and its incidence plateaus by three years of age [2,4,6].

The functional role of PBMC HLA-DQ cell surface expression in relation to the appearance of a first autoantibody in children at high genetic risk of type 1 diabetes is poorly understood. It was reported in monozygotic twins that CD8^+^ T cells expressed HLA-DR prior to clinical onset [9]. However, recent data suggest that the association between HLA-DR-DQ is mainly with the first appearing autoantibody [2,5,6], rather than with the autoantibodies appearing later [5]. After the first appearance of IAA, GADA, or both, about 60% of the children develop a second autoantibody within one year, and 70% of children with multiple persistent autoantibodies will progress to clinical diabetes [1,6], and more than 70% of those children will go on to a clinical diagnosis of type 1 diabetes within 10 years from seroconvesion.

Despite persistent antigen stimulation, maintaining T cell function contribute to the ability to clear chronic infection and cancer. Maintaining robust responses can prove harmful rather than helpful when T cell immunity targets self antigen or excessively damages healthy tissues [10]. In the mechanism of T cell exhaustion, where cellular immunity is regulated, CD8^+^ T cells lose effector ability during the response to persistent stimulation [10,11]. An example of immune system reactivity to persistent self-antigen is that T cell exhaustion has been inversely associated with a relapsing course of autoinflammatory disease [10]. The persistence of antigen and lack of accessory ‘help’ signals have been found to drive the development of CD8 T cell exhaustion during chronic infection [11]. In autoimmunity, pronounced CD4 T cell co-stimulation has been observed together with reduced CD8 T cell exhaustion [11]. So far, progression rather than onset of autoinflammatory disease has been associated with the process of T cell exhaustion [10].

The aim of this study was to test the hypothesis that the expression of HLA-DQ on peripheral blood cells in a cross-sectional analysis was associated either with the number of autoantibodies at 10–15 years of age or with autoimmunity burden defined as the overall exposure to autoantibodies during follow-up. Hence, our 67 research subjects were analyzed both in a cross-sectional sample and as a cumulative area under the trajectory of autoantibodies during follow-up. To our knowledge, this is a first exploratory study of children at increased risk of type 1 diabetes first followed longitudinally and then subjected to a cross-sectional analysis to determine the cell surface expression of HLA-DQ on peripheral blood cells. It has been reported that persistent elevation of HLA-DR^+^ CD8^+^ T-cells was found prior to clinical onset [9] but expression of HLA-DQ has to our knowledge not been reported previously. As HLA-DQ is strongly associated with the genetic risk not only for type 1 diabetes [12] but primarily for the first appearing beta cell autoantibody [2,3], we analyzed HLA-DQ cell surface expression in sorted peripheral blood cells in relation to HLA-DQ determined by Next Generation Sequencing (NGS) and four different autoantibodies.

The paper is organized as follows: first, we describe the study sample and the methods used to analyze the blood samples collected as part of this study, next we describe the statistical methods used to describe our study sample and test our hypotheses. In order to explore the relationship between HLA-DQ cell surface expression on peripheral blood cells and the exhaustion of immune cells, we consider the following hypotheses: whether the expression of HLA-DQ on peripheral blood cells is associated with autoimmunity burden and if HLA-DQ cell surface expression varies with autoimmunity burden.

## 2 Materials and methods

### 2.1 Compliance with ethical standards

The DiPiS study was intitated in 2000. It complied with ethical standards and did not pose a significant risk for the research subjects. All procedures performed in studies involving human participants were in accordance with the ethical standards of the institutional and/or national research committee (the Regional Ethical Review Board in Lund: Dnr 2009/244, Dnr 2014/196 and Dnr 2015/861) and with the 1964 Helsinki declaration and its later amendments or comparable ethical standards. Blood sampling was preceded by EMLA cream to reduce pain, The research subjects have signed informed consent to participate in the present cross-sectional follow-up investigation.

### 2.2 Study participants

A blood sample (30–50 mL) was obtained from 67 children at an increased risk of type 1 diabetes followed in the DiPiS study [[13], [14], [15]]. DiPiS is a prospective population-based cohort study with the aim to investigate the genetic and environmental factors that might contribute to, or trigger, the development of type 1 diabetes [14,[16], [17], [18]]. Briefly, newborn children (n = 35 683) in the general population were screened and asked to participate in the study if any of the following conditions were satisfied: if they had a high-risk HLA based on their HLA-DQ genotype, had a first degree relative (FDR) with type 1 diabetes, any autoantibodies were detected in cord blood, the mother reported having had an infection or gestational diabetes during pregnancy [14,18]. The selected children (n = 3889) were followed from 2 years of age either annually (negative or single autoantibody) or every three months (if multiple autoantibodies were detected at any earlier visit) until the age of 15 or diagnosis of type 1 diabetes, whichever occurred first. From the subset of DiPiS children still under follow-up, not diagnosed with type 1 diabetes, children (n = 98) between ages 10 and 15 were selected at random and contacted, of those, 67 agreed to donate blood. The children have provided capillary blood samples, often from home, for autoantibody analyses at least 4 times during follow-up and as many as 33 times until our venous blood sampling. Because of random selection, autoantibody negative children with high risk HLA were overrepresented in our cohort, as children with high risk HLA and single or multiple autoantibodies were likely to have already been diagnosed with type 1 diabetes.

Blood glucose and HbA1c were available for all children in intense follow-up (2 or more autoantibodies simultaneously), the measurements were normal (random p-glucose < 11.1 mmol/L and HbA1c ranged between 27 and 40 [19]) and these children would therefore be classified as stage 1 type 1 diabetes according to the current nomenclature [20].

### 2.3 Beta cell autoantibodies

IAA, GADA, insulinoma-associated protein-2 (IA-2A), and three variants of zinc transporter 8 autoantibodies (ZnT8A) against arginine, tryptophan or glutamine at position 325 (R/W/Q, respectively) were measured annually or quarterly as part of the DiPiS protocol, and at the sampling for our study. IAA was first screened in a non-competitive assay followed by a competitive assay adapted for microtiter plate format as previously described [21]. Levels (Units/mL) of GADA, IA-2A and ZnT8A were determined, using in-house verified threshold values, in serum or plasma as previously described [14,[22], [23], [24]]. Duplicate samples of serum or plasma were incubated overnight at 4^ᵒ^C with [^35^S]methionine-labelled antigens. Free labelled antigen was separated from antibody bound by Protein A-Sepharose (PAS, catalog number 101090, Invitrogen, Carlsbad, California, USA). Bound radioactivity was determined in a β-counter (2450 Microplate Counter, PerkinElmer, Waltham, Massachusetts, USA) and levels expressed in U/mL using in-house standards. In the 67 children, autoantibodies in 24 children were determined in plasma from blood diluted 1:1 in RPMI 1640 while in 43 children autoantibodies were determined both in plasma and in RPMI 1640 diluted blood.

Autoantibody status was defined as negative if the level of autoantibodies was less than or equal to 34 U/mL (GADA), 5 U/mL (IA-2A), 0.79 U/mL (IAA), 59 U/mL (ZnT8A triple assay), 63 U/mL (ZnT8RA), 74 U/mL (ZnT8WA) and 99 U/mL (ZnT8QA). Levels of ZnT8A were adjusted for sample storage duration resulting in a second threshold value for samples stored up to or longer than 3.5 years, as concentrations increased with increasing storage time at 20 °C. For ZnT8A the autoantibody status in samples stored longer than 3.5 years was defined as negative if the level of autoantibodies was less than or equal to 129 U/mL (ZnT8RA), 99 U/mL (ZnT8WA) and 149 U/mL (ZnT8QA). The cut-off values were based on the 97.5th percentile of healthy blood donors [14,22,25].

The intra-assay and inter-assay coefficients of variation, respectively, were 6.9% and 8.5% for GADA, 9.8% and 6.4% for IA-2A, 10.0% and 11.6% for screening of IAA, 7.4% and 6.8% for IAA COMP, 9.8% and 1.2% for ZnT8RA, 9.8% and 4.4% for ZnT8WA, and 9.6% and 5.0% for ZnT8QA.

Our laboratory is participating in the Islet Autoantibody Standardization Program (IASP). At the 2018 serum exchange, sensitivity and specificity was set at 64.0% and 94.5%, respectively, for GADA; 62.0% and 100.0%, respectively, for IA-2A; 18.0% and 96.7%, respectively, for IAA; 40.0% and 100.0%, respectively, for ZnT8RA; 54.0% and 100.0%, respectively, for ZnT8WA; as well as 52.0% and 100.0%, respectively, for ZnT8QA.

### 2.4 Blood sampling and complete blood count

In the present protocol, we obtained a large volume of blood (30–50 mL) from 67 DiPiS children. The subjects were non-fasting and a blood sample was collected in five 10 mL BD Vacutainer Sodium Heparin tubes with Green Conventional Closure (catalog number 368480, Becton Dickinson, Franklin Lakes, New Jersey). Whole blood (300 μL) was used for complete blood count (CBC) using a multi-parameter automated hematology analyser (CELL-DYN®, Ruby Hematology Analyser, Abbott Diagnostics, Lake Forest, Illinois). Number of cells (10^9^ cells/μL) were used to monitor isolation of cells following cell fractionation.

### 2.5 Isolation of peripheral blood cells

The peripheral blood cell extraction protocol was adapted from Ref. [26], a protocol followed as cells were intended for bulk epigenetics analysis [27]. Briefly, the five 10 mL blood samples were pooled at room temperature at equal volumes into two 50 mL Falcon tubes and diluted 1:1 in RPMI 1640 Medium (GlutaMAX^TM^ Supplement, HEPES, Invitrogen) supplemented with 1% (v/v) penicillin-streptomycin and 0.2% (v/v) Bip-Pure Human Serum Albumin (catalog number 05-720-1B, Biological Industries, Cromwell, Connecticut, USA). The tubes were left rolling overnight at room temperature. Ficoll-Hypague (catalog number 17-1440-02, GE Healthcare, Little Chalfont, United Kingdom) was used to prepare PBMC. The red blood cell pellet was resuspended in PBS supplemented with 13 mM TriSodium Citrate to lyse the red blood cells and rescue granulocytes. CD19^+^ B cells, CD16^+^CD66^+^ neutrophils, CD16^+^ cells, CD14^+^CD16^−^ classical monocytes, CD4^+^ helper T cells and CD8^+^ cytotoxic T cells were isolated using MicroBeads conjugated with mouse monoclonal anti-human antibodies and MACS according to the instructions by the manufacturer (Miltenyi Biotec, Bergisch Gladbach, Germany).

### 2.6 Flow cytometry

Flow cytometry was carried out on an CyAnADP® (Beckman Coulter, Brea, California, USA) using the Summit v4.3 software (DAKO, Copenhagen, Denmark) for each of the isolated cell subsets. Isolated cell subsets were stained with titrated monoclonad antibodies (Table 1A), adapted from Ref. [26]. The HLA-DQ antibody clone REA303 recognizes the same epitopes as the pan DQ-specific SPV-L3. Fluorescence minus one (FMO) controls used in analysis of neutrophils and monocytes are presented in Tables 1B and 1C, respectively. Stained and unstained samples of PBMC (original, not sorted) and erythrocyte pellet were used as controls to distinguish between non-stained and stained PBMC (B cells, CD16 cells, monocytes and T cells) and neutrophils respectively. Cells were fixated in 4% (v/v) formaldehyde and 0.01% (v/v) sodium azide in PBS and analyzed within seven days (median: 3 days) of fixation. The instrument was calibrated prior to each run using UltraComp eBeads (catalog number 01–2222, Affymetric eBiosciences, Santa Clara, California, USA).

**Table 1:** **Flow cytometry antibodyies used in staining of isolated peripheral blood cells**. Antibody panel (a) for the individual cell types, the specific markers, fluorescent marker and clone. Antibodies were titered for optimal staining of cells. Setting up of the FMO controls for analyzing neutrophils (b) and monocytes (c), where the tubes are represented by the first column and the rest of the columns specify which antibody to add in a tube. For example, the FITC FMO in neutrophils will include the PE, and PerCP-Cy5.5 but not the FITC antibody. In neutrophils, a few sample were stained with CD66b BB515 instead of CD66b FITC because of ordering and shipping trouble, this should not affect the purity assessment since all samples are individually analyzed with their own FMO controls.

A			
Cell subset	Specific marker	Fluorescent marker	Clone
Neutrophils	CD66b CD16 CD45	FITC/BB515 PE PE-Cy5.5	BIRMA 17C/REA306 VEP13 H130
B cells	CD19	PE	LT19
CD16^+^ cells	CD16	PE	B73.1/leu11c
Monocytes	CD14 CD16 CD64 CD45	FITC PE PerCP-Cy5.5 PE-Cy7	MφP9 B73.1/leu11c 10.1 HI30
CD4 T cells	CD4	FITC	M-T466
CD8 T cells	CD8	FITC	LT19
Individual cell cubsets	HLA-DQ	FITC/BB515	REA303

B			
	FITC	PE	PE-Cy5.5
FITC	–	✓	✓
PE	✓	–	✓
PE-Cy5.5	✓	✓	–

C				
	FITC	PE	PerCp-Cy5.5	PE-Cy7
FITC	–	✓	✓	✓
PE	✓	–	✓	✓
PerCp-Cy5.5	✓	✓	–	✓
PE-Cy7	✓	✓	✓	–

Acquired data was analyzed with Kaluza Analysis Software 1.5a (Beckman Coulter) for purity of the isolated cell subsets as well as HLA-DQ cell surface MFI. Gating strategy for identifying cell populations and HLA-DQ cell surface MFI on the isolated cell subsets was adapted from Ref. [26] and illustrated in Supplemental Fig. 1. First, duplicate events were removed using scatterplot of FS Area and FS. Cell populations were identified in the initial gate using Side scatter (SSC) and forward scatter (FSC). Up to 10 000 events were recorded. The cell population was plotted in a histogram using the appropriate cell specific or HLA-DQ antibody for identification of cells and HLA-DQ fluorescence intensity, respectively. Unstained, not sorted, PBMC or erythrocyte pellet were used as negative controls in the plot.

Data of HLA-DQ cell surface MFI from six subjects, where an APC fluorescent marker was used, is not comparable with the FITC fluorescent marker. These six subjects were therefore excluded from the analyses.

### 2.7 HLA Next Generation Sequencing

Dried blood spot 6 mm punch-outs were sent blinded to Cisco Systems Inc. (Seattle, USA) to perform NGS of HLA Class II -*DRB345*, -*DRB1*, -*DQA1*, -*DQB1, -DPA1* and -*DPB1*. Briefly, HLA NGS utilizes PCR-based HLA amplification and sequencing with Illumina MiSeq technology as described [28,29]. Extended HLA haplotypes were determined from the allelic information, an online database (Allele Frequencies in Worldwide Population, http://www.allelefrequencies.net) [30].

### 2.8 Statistical analyses

Autoantibody frequency for GADA, IAA, IA2A, ZnT8RA, ZnT8WA and ZnT8QA were available for all visits in the DiPiS follow-up. These were dichotomized into autoantibody status: positive if the titer for a given autoantibody was greater than its associated threshold and negative otherwise. Measurements of the following variables were made at the time of sampling into this study: CBC, HLA-DQ cell surface MFI as measured by flow cytometry for the isolated subtypes of peripheral blood cells (CD16^+^CD66^+^ neutrophils, CD19^+^ B cells, CD16^+^ cells, CD14^+^CD16^−^ classical monocytes, CD4^+^ T cells and CD8^+^ T cells).

We considered two measures as proxies for *autoimmunity burden* – sampling Autoimmunity Burden (sAB) and cumulative Autoimmunity Burden (cAB), (based on DiPiS follow-up from 2 years of age until the time of sampling). To estimate the sAB, we calculated the number of autoantibodies present at the time of sampling, counting GADA, IAA and IA2A as 1 if positive and 0 otherwise, and counting ZnT8A as 1 if any of ZnT8WA, ZnT8RA and ZnT8QA were positive. These were grouped into negative, single or multiple (0, 1 and 2+, respectively) autoantibodies and treated as a factor in the analyses. The cAB was estimated as the area under the trajectory of autoantibodies over time in, and stratified into tertiles (low, medium, high = (0–4.61], (4.61–13], (13–45], respectively). Additional variables used in the analyses were age at sampling, gender, HLA-DQ2/8 genotype based on NGS (1 if HLA-DQ2/8, 0 otherwise).

We describe the data sample in terms of demographic characteristics, autoantibody frequency, and CBC stratified by autoantibody group (0/1/2+ or low/medium/high) and NGS haplotypes (Supplemental Table 1). We used boxplots of HLA-DQ cell surface MFI stratified by autoimmunity burden (sAB and cAB, as factors) and likelihood ratio tests to examine the association between HLA-DQ cell surface expression and autoimmunity burden for each cell type. Similarly, we used boxplots and likelihood ratio tests to examine the association between CBC and autoimmunity burden (sAB and cAB). Histograms of CBC and HLA-DQ MFI on each of the isolated cell subsets were used to assess their distributions and identify possible outliers.

To examine the association between HLA-DQ cell surface MFI and autoimmunity burden we fit linear models with the HLA-DQ cell surface MFI as the outcome, with autoimmunity burden as the predictor (separate models for sAB and cAB), adjusting for age at sampling, sex, and HLA-DQ2/8; and additionally adjusting for CBC (white blood cells, red blood cells, and platelets). Standard errors were estimated using robust methods, as well as model-based as a sensitivity analysis. To determine whether autoimmunity burden is a mediator of the association between HLA and HLA-DQ cell surface MFI, we fit the same models excluding autoimmunity burden.

The analysis was performed in R version 3.6.1 (http://www.r-project.org). Benjamini-Hochberg procedure [31] was used to control the false discovery rate at 5%. P-values presented are nominal and those that remain significant after adjustment for multiple comparisons are indicated by an asterisk. P-values that remain significant after the multiple comparison adjustment are considered to be statistically significant. Nominal p-values < 0.005 are considered to be suggestive of an association.

## 3 Results

**Fig. 1:**
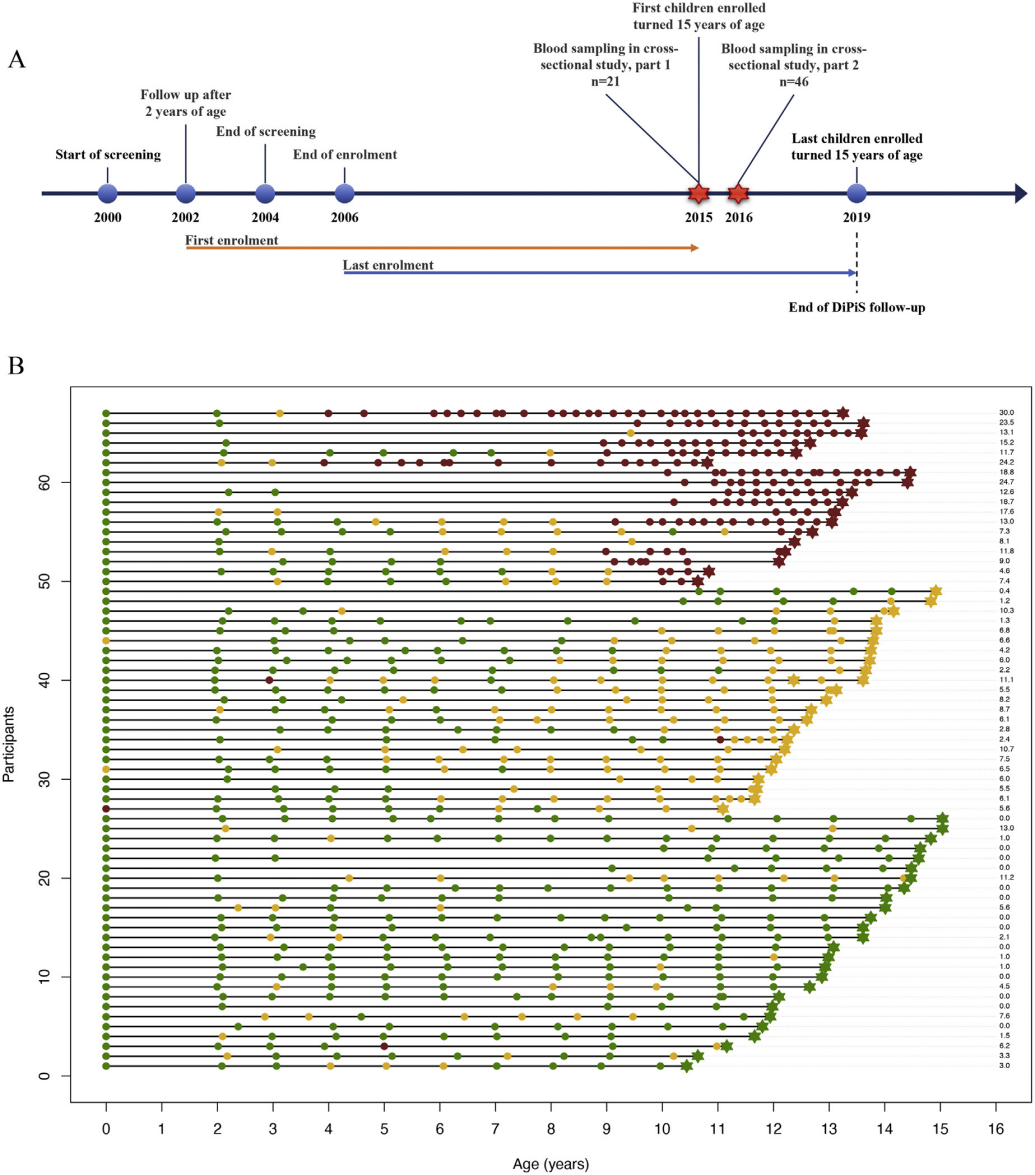
**Schematic of the Diabetes Prediction in Skåne (DiPiS) study timeline and time of sampling into our cross-sectional study.** A timeline (panel A) of the key events of enrolment and follow-up in DiPiS and sampling in the present study. Screening of high risk for type 1 diabetes was carried out from 2000 until 2004. Children with increased risk of type 1 diabetes were enrolled at 2 years of age between 2002 and 2004 and followed either annually (negative or single autoantibody) or every three months (if multiple autoantibodies were detected at any earlier visit) until the age of 15 or diagnosis of type 1 diabetes. The sampling into our study was divided in two parts, part 1 where n = 21 subjects were sampled and part 2 where n = 46 subjects were sampled. Autoantibody profiles (panel B) of the n = 67 children in our study during follow-up as part of the DiPiS study and at time of sampling into our study. The timeline plot shows the visits (circles for visits as part of DiPiS follow-up, stars for time of sampling into our study) and autoantibody count (negative = green, single = yellow, multiple = red). Autoantibodies measured were GADA, IA2A, IAA and any of the three variants of ZnT8A against arginine, tryptophan or glutamine at position 325 (R/W/Q, respectively). Autoimmunity burden calculated as area under the trajectory of autoantibodies over time is presented to the right.

The DiPiS study timeline and sampling are shown in Fig. 1A, and the data available for our study, both the historical follow-up as part of DiPiS and time of sampling into our study are shown in Fig. 1B. The subjects were stratified by sAB and sorted according to an increasing follow-up time in DiPiS within the strata, with the cAB level displayed for each subject. Individual autoantibody profiles during follow-up, shown in Supplemental Fig. 2, demonstrate a highly variable pattern of first appearing autoantibody and later developing multiple autoantibodies in some of the children. The demographic characteristics, HLA, and autoantibody level information, stratified by sAB and cAB (Tables 2A and 2B, respectively) were similar across the three levels of autoimmunity burden. HLA-DQ2/8 genotype differed across the levels, with the highest proportion of HLA-DQ2/8 children in the lowest autoimmunity burden strata. Detailed HLA information for all subjects based on NGS is shown in Supplemental Table 1.

**Table 2:** **Characteristics of our study sample stratified by autoimmunty burden measured as the number of autoantibodies detected at the time of sampling (panel A) or cumulative autoimmunity burden (cAB) measured as area under the trajectory of autoantibodies over time (panel B).** The children (n = 67) participating in this study were screened for type 1 diabetes risk at birth and enrolled in the DiPiS (Diabetes Prediction Study in Skåne) study at 2 years of age and followed annually if remaining negative or quarterly if they had a single or multiple autotantibodies, respectively.

**A**			
**Study subjects (n = 67)**	**0 autoantibodies (n = 26)**	**1 autoantibody (n = 23)**	**2+ autoantibodies (n = 18)**
Females, n (%)	14 (53.8)	11 (47.8)	10 (55.6)
Age (years), mean (sd)	13.2 (1.38)	13.0 (1.07)	12.7 (1.11)
Follow-up time, mean (sd)	10.4 (2.09)	10.0 (2.06)	8.75 (2.89)
Follow-up visits, mean (sd)	10.3 (2.84)	10.8 (2.53)	14.5 (6.14)
HLA-DQ2/8, n (%)	25 (96.2)	12 (52.2)	4 (22.2)
*Median levels (interquartile ranges) of autoantibody titers (U/mL) at sampling*			
GADA	9.6 (7.8, 14.5)	93.0 (39.0, 217.0)	152.0 (52.2, 730.0)
IA-2A	1.1 (0.8, 1.7)	1.6 (1.2, 1.8)	112.0 (22.4, 902.0)
IAA	0.07 (0.00, 0.17)	0.17 (0.00, 0.30)	1.52 (0.37, 3.18)
ZnT8RA	12.2 (8.9, 16.3)	13.6 (10.7, 16.6)	36.0 (17.5, 114.0)
ZnT8WA	15.8 (8.5, 21.3)	13.7 (7.8, 19.8)	20.1 (4.2, 73.6)
ZnT8QA	17.0 (7.4, 21.3)	16.7 (11.1, 19.5)	23.9 (16.3, 104.0)

**B**			
**Study subjects (n = 67)**	**Low [0, 4.61] (n = 29)**	**Medium (4.61, 13] (n = 29)**	**High (13, 45] (n = 9)**
Females, n (%)	17 (58.6)	13 (44.8)	5 (55.6)
Age (years), mean (sd)	13.2 (1.33)	12.7 (1.08)	13.2 (1.08)
Follow-up time, mean (sd)	9.9 (2.47)	10.4 (1.25)	7.6 (3.66)
Follow-up visits, mean (sd)	10.9 (2.58)	11.0 (3.81)	15.9 (7.15)
HLA-DQ2/8, n (%)	27 (93.1)	12 (41.4)	2 (22.2)
*Median levels (interquartile ranges) of autoantibody titers (U/mL) at sampling*			
GADA	11.5 (8.8, 31.3)	50.4 (13.3, 260.0)	423.0 (57.5, 736.0)
IA-2A	1.3 (0.8, 1.7)	1.7 (1.1, 3.9)	756.0 (68.1, 2840.0)
IAA	0.05 (0.00, 0.16)	0.25 (0.10, 0.50)	1.72 (1.31, 2.02)
ZnT8RA	13.4 (8.9, 18.7)	13.1 (10.2, 19.2)	56.2 (17.9, 104.0)
ZnT8WA	17.0 (8.9, 24.3)	11.0 (2.5, 19.8)	25.0 (17.6, 59.8)
ZnT8QA	17.7 (8.5, 21.0)	16.9 (11.5, 23.5)	23.8 (14.0, 67.4)

The follow-up time ranged from 10.5 to 15.0 years. Cord blood for three children was autoantibody positive. Two of these children had a mother with type 1 diabetes, while one child was born to a non-type 1 diabetes autoantibody-positive mother. The first follow-up sample at two years of age showed that 7 children had already developed a single autoantibody. At the time of sampling into the present study, 13 children were autoantibody negative at any of the times measured, 12 subjects had lost one autoantibody and became autoantibody negative, 23 had a single autoantibody and 18 had mutliple autoantibodies (Fig. 1). Also, 7 children with multiple autoantibodies lost one (n = 5) or two (n = 2) autoantibodies during follow-up.

**Fig. 2:**
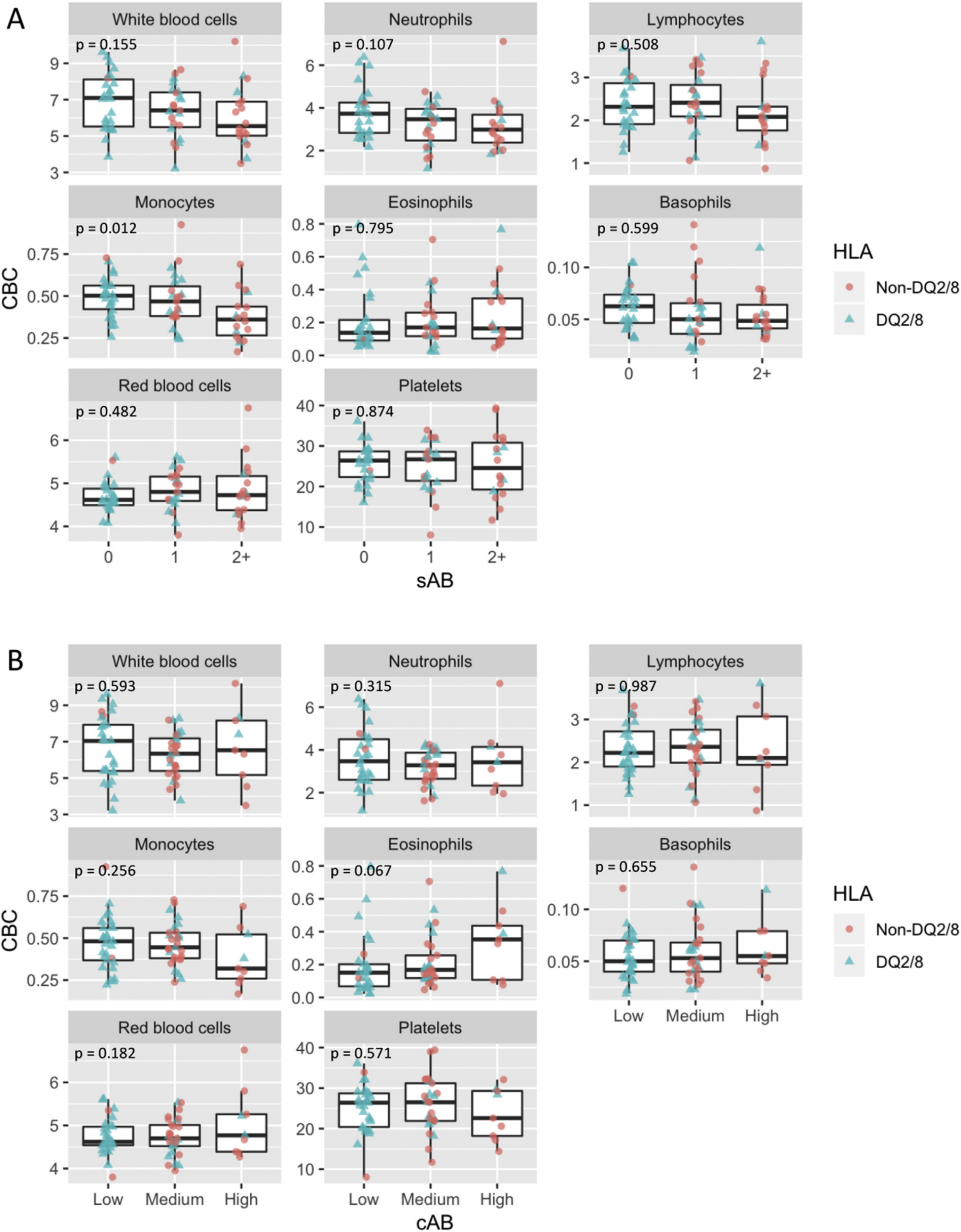
**Complete blood count of whole blood in n = 67 children, stratified by the autoimmunity burden measured as the number of autoantibodies detected at the time of sampling (sAB) (panel A) or cumulative autoimmunity burden (cAB) measured as area under the trajectory of autoantibodies over time (panel B).** The HLA-DQ2/8 status is indicated by the color and shape (blue triangles: DQ2/8, red circles: non-DQ2/8). P-value (p) shown is the nominal p-value obtained using likelihood ratio tests to examine the association between complete blood count and sAB or cAB, respectively, for each cell type. None of the p-values remained significant after adjustment for multiple comparisons (corrected using the Benjamini-Hochberg procedure assuming 16 comparisons and a 5% false discovery rate).

CBC levels stratified by autoimmunity burden, sAB and cAB, are shown in Fig. 2, panels A and B, respectively. In the CD14^+^CD16- classical monocytes we noted a modest decline of CBC with increasing sAB (p = 0.012), but we did not observe a similar association with cAB (p = 0.256). The levels of CBC appear stable across autoimmunity burden strata for the other cell types.

**Fig. 3:**
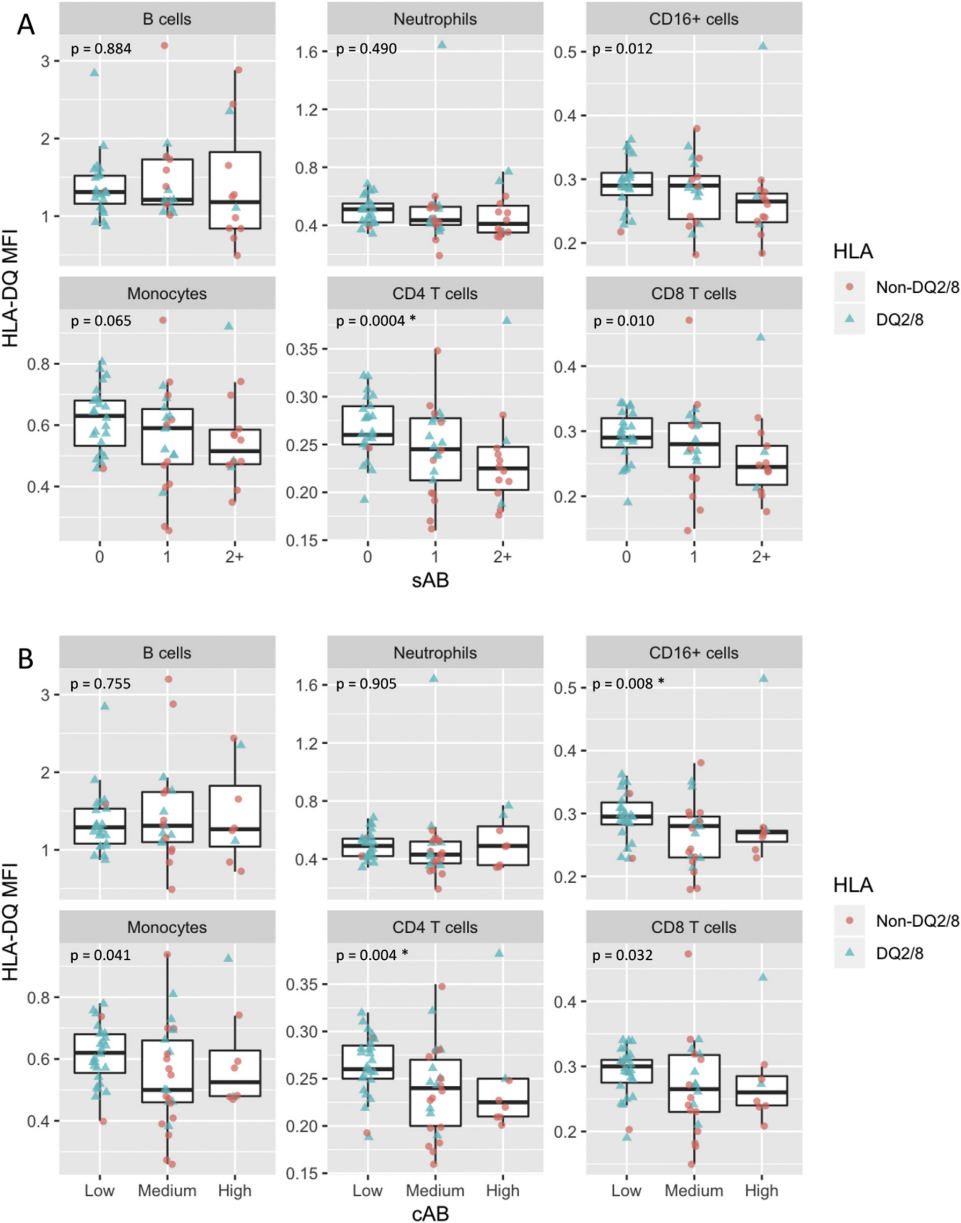
**HLA-DQ cell surface median fluorescence intensity (MFI) on isolated peripheral blood cells in n = 67 children, stratified by autoimmunity burden measured as the number of autoantibodies detected at the time of sampling (sAB) (panel A) or cumulative autoimmunity burden (cAB) measured as area under the trajectory of autoantibodies over time (panel B).** The HLA-DQ2/8 status is indicated by the color and shape (blue triangles: DQ2/8, red circles: non-DQ2/8). P-value (p) shown is the nominal p-value obtained using likelihood ratio tests to examine the association between HLA-DQ cell surface MFI and sAB or cAB, respectively, for each cell type. The p-values that remain significant after adjustment for multiple comparisons are indicated with a ∗ (corrected using the Benjamini-Hochberg procedure assuming 12 comparisons and a 5% false discovery rate).

HLA-DQ cell surface MFI stratified by autoimmunity burden is shown in Fig. 3A for sAB and 3B for cAB. The MFI declined with increasing sAB for CD4^+^ T cell (p = 0.0004), and marginally for CD16^+^ cells (p = 0.012) and CD8^+^ T cells (p = 0.010). A similar trend was seen for HLA-DQ cell surface MFI stratified by cAB, with the MFI declining in CD4^+^ T cells (p = 0.004), and CD16^+^ cells (p = 0.008).

**Fig. 4:**
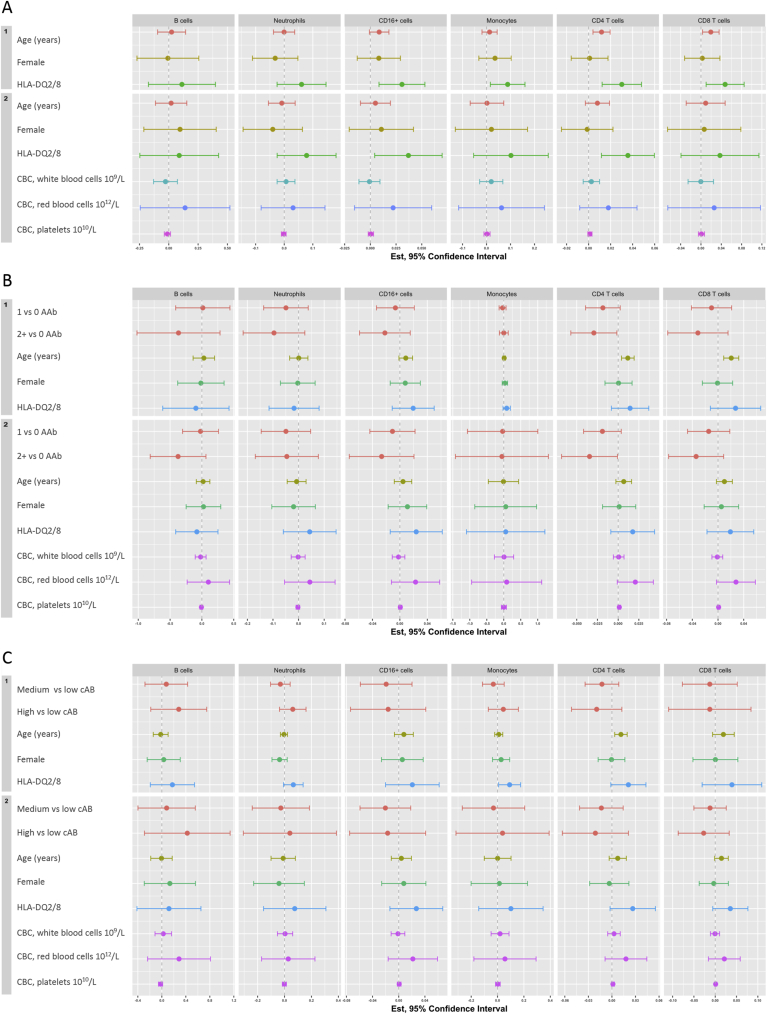
**Estimates and 95% confidence intervals (Est, 95% CI)) of the association between HLA-DQ cell surface median fluorescence intensity (MFI) on isolated peripheral blood cells and HLA-DQ2/8 (panel A) and with autoimmunity burden measured as the number of autoantibodies detected at the time of sampling (sAB) (panel B) or cumulative (cAB) measured as area under the trajectory of autoantibodies over time (panel C)**. The models for HLA-DQ2/8 were adjusted for age and sex (Model 1) and complete blood count (CBC) of peripheral blood cells, red blood cells and platelets in addition to the parameters in Model 1 (Model 2). The models were fit using linear regression with robust standard errors. (See the Supplemental Table 2 panel A–C, respectively, for detailed results corresponding to these plots).

The association between HLA-DQ2/8 and HLA-DQ cell surface MFI on six sub-types of isolated peripheral blood cells was analyzed in two models each for 1) without adjusting for autoantibodies (Fig. 4A and Supplemental Table 2A); 2) adjusting for sAB (Fig. 4B and Supplemental Tables 2B) and 3) adjusting for cAB (Fig. 4C and Supplemental Table 2C). The estimates from the two models and their associated 95% confidence intervals were based on linear regression with robust standard errors and were additionally adjusted for age at sampling and sex (model 1) plus CBC (model 2). The corresponding results for linear regression using model-based standard errors are shown in Supplemental Fig. 3 and Supplemental Tables 2D–F.

In model 1, without adjusting for autoantibodies, HLA-DQ cell surface MFI measured in children with the HLA-DQ2/8 genotype compared to non-HLA-DQ2/8 children was 0.031 ((0.008, 0.053) p = 0.009) higher in CD16^+^ cells; 0.030 ((0.012, 0.048) p = 0.002) higher in CD4^+^ T cells; it was marginally higher in CD14^+^CD16^−^ classical monocytes and CD8^+^ T cells (Fig. 4A and Supplemental Table 2A).

In model 2, without adjusting for autoantibodies, HLA-DQ cell surface MFI was 0.036 ((0.012, 0.060) p = 0.005) higher in CD4^+^ T cells for HLA-DQ2/8 compared to non-HLA-DQ2/8 children; it was marginally higher in CD16^+^ cells (Fig. 4 and Suplemental Table 2A).

After additionally adjusting for sAB (Fig. 4B and Supplemental Table 2B), or cAB (Fig. 4C and Supplemental Table 2C), in both model 1 and 2, the association between HLA-DQ2/8 and cell surface HLA-DQ cell surface MFI was no longer observed.

We observed an association between age and HLA-DQ MFI in CD4^+^ T cells in model 1 (adjusting for sex and HLA-DQ2/8) but not in model 2 (additionally adjusting for CBC) (Supplementary Table 2A). Similarly, the association with age also remained in model 1, but not model 2, after adjusting for sAB (Supplemental Table 2B) or cAB (Supplemetal Table 2C).

The results based on linear regression with robust standard error estimation shown above were consistent with the results based on linear regression models with model-based standard error estimation presented in Supplemental Fig. 3 and Supplemental Table 2 D-F.

## 4 Discussion

In this unique cross-sectional study of 67 children randomly selected from the DiPiS study [16,18], we wanted to test the hypothesis that exposure to autoantibodies was associated with the peripheral blood cell HLA-DQ cell surface expresson. This was a reasonable hypothesis as HLA-DQ is strongly associated with not only the risk for type 1 diabetes [12,19] but also with the first appearing beta cell autoantibody [2,4]. As the 67 children had been regularly followed from birth, with the first beta cell autoantibody test performed at 2 years of age, and then examined 10–15 years later, it was possible to test the hypothesis in two ways. First, we tested whether the HLA-DQ cell surface MFI was associated with the autoimmunity burden at sampling (sAB), second, we tested whether it was associated with the cumulative autoimmunity burden during follow-up (cAB). The major finding that HLA-DQ cell surface MFI decreased with increasing autoimmunity burden in both sAB and cAB, strongly suggests that the presence of autoantibodies affects the ability of some peripheral blood cells to present antigen. It is speculated that a reduction in HLA-DQ class II antigen expression, for example, may reduce the ability to maintain peripheral tolerance [32,33]. As expected, neutrophils showed negligible fluorescence, while B cells (CD19^+^) and monocytes (CD14^+^CD16^−^) showed significant fluorescence (Supplemental Fig. 1). It is, therefore, notable that the autoimmunity burden, be it sAB or cAB, was associated with a decreased HLA-DQ fluorescence intensity in monocytes, but not in B cells.

HLA-DQ cell surface expression on CD4^+^ and CD8^+^ T cells has been documented in health [34] as well as in relation to both autoimmune conditions (for example T1D, CD and vitiligo) and infectious episodes [35,36]. Our observation that HLA-DQ cell surface MFI was decreased both with increasing number of autoantibodies (sAB) as well as with cumulative exposure (cAB) suggests that sAB and cAB play a role of mediator of the association between HLA-DQ2/8 and HLA-DQ cell surface expression measured by MFI. The association between HLA-DQ2/8 and cell surface HLA-DQ MFI was no longer observed after adjusting for sAB or cAB, suggesting that autoimmunity burden plays a role of a mediator in that association.

As far as we know, the *cumulative autoimmunity burden* remains to be fully established as is a standard approach to measure it. For the purposes of this manuscript, our aim was to quantify that burden, i.e. the burden of autoantibodies over time, and estimating the area under the trajectory of the autoantobodies over time was the natural choice. It is well established that HLA-DQ2/8 contributes the highest risk for type 1 diabetes and the risk for progression to clinical onset increases with increasing number of autoantibodies [1,37]. Therefore we believe that the burden of autoantibodies over time puts a strain on the subject and could possibly contribute to T cell exhaustion. T cell exhaustion is thought to allow partial containment of chronic infections by persistence of T cells, without causing immunopathy [38,39]. It has been suggested that T cell exhaustion may be of importance to limit immunopathology or autoreactivity [38,40] and it cannot be excluded that our observation that the decrease in HLA-DQ cell surface MFI by sAB and cAB is related to T cell exhaustion.

A strength of the present study is the availability of autoantibody data from longterm follow-up of children randomly selected from the DiPiS study. In addition, it was possible to obtain comprehensive data on HLA by NGS, autoanibodies, CBC, and HLA-DQ cell surface MFI at the time of sampling. None of the children had diabetes at the time of sampling. At the time of writing, 5 children have been diagnosed with diabetes after sampling (6.0, 8.7, 9.6, 11.3 and 26 months after sampling). Therefore, the current research subjects represent children at various stages of the pathogenesis. It is noted that HLA-DQ2/8 children in this cohort are mostly autoantibody negative. This is not surprising as 3.5% of newborns have this genotype and only a fraction of such children will develop one or several autoantibodies let alone autoimmune type 1 diabetes. It is also possible that some DiPiS HLA-DQ2/8 children have already been diagnosed with type 1 diabetes and therefore not asked to participate in the present investigation.

A weakness of our study is the lack of cellular analyses during follow-up, which was not possible to accomplish for reasons of resources, place of residence and logistics. Another potential weakness is the use of a pan-HLA-DQ monoclonal antibody, it would have been of interest to use allele specific HLA-DQ antibodies, unfortunately, such antibodies were not available. However, the pan-HLA-DQ antibody used allowed us to test the hypothesis that HLA-DQ expression was related to sAB and cAB.

To the best of our knowledge autoimmunity burden has previously not been considered in studies of children who have been beta cell autoantibody positive from an early age. A study in monozygotic twins demonstrated that the pathogenesis prior to clinical onset was characterized by persistent elevation of HLA-DR^+^ CD8^+^ T-cells [9]. At the time of sampling all our study subjects were healthy and all had HbA1c and glucose levels within the normal range. However, the subjects had been positive for autoantibodies between 3 and 13 years. The current understanding of the beta cell autoimmune pathogenesis is poor but several follow-up studies reveal a heterogenous pattern. Progression to clinical onset may be faster in children with seroconversion early in life [1]. The immune system in young children is constantly trained by infections for an adaptive immune response and thus an exhaustion of immune cells could potentially speed up the progression to diabetes. Immune exhaustion refers to immune dysfunction, poor effector function of immune cells, due to autoimmune burden (reviewed in Refs. [10,11]). Relative levels of T-cell exhaustion have been shown to be associated with clinical outcome in chronic viral infection [40].

The length of the prodrome period, time from the appearance of multiple autoantibodies until the diagnosis of diabetes, is inversely proportional to the number of autoantibodies: the more autoantibodies, the faster the rate of progression to clinical onset [1,41,42]. Genetic, primarily non-HLA genetic factors, in addition to the beta cell autoantibody markers and environmental factors [43,44] also seem to increase the rate of progression of disease. Thus, the number of autoantibodies is a strong predictive marker of the pathogenesis. However, it is still unclear when mononuclear cells begin to invade the pancreatic islet in beta cell autoantibody positive subjects. It has been reported that pancreas organ donors with beta cells autoantibodies are negative for insulitis which was only found in donors with multiple autoantibodies [45,46]. In other autoimmune diseases such as Crohn's disease and systemic lupus erythematosus autoreactive mononuclear cells target the end organ and T cell exhaustion is easily demonstrated (reviewed in Refs. [10,11]). Disease-related signatures were identified by the use of gene expression and transcriptional profiling of PBMC [47]. While we have defined autoimmunity burden as exposure to autoantibodies it can only be speculated to what extent reduced HLA-DQ cell surface expression on T cells and monocytes reflects the type of T cell exhaustion reported in other autoimmune diseases. HLA-DQ is constitutively expressed on monocytes and activated T cells express HLA Class II heterodimers [9]. Further studies are therefore warranted to determine whether children with multiple autoantibodies exhibit T cell exhaustion as defined by poor effector function, sustained expression of inhibitory receptors or a transcriptional pattern different from that of functional effector or memory T cells (reviewed in Ref. [10,11]).

Variation in HLA-DQ cell surface expression on six types of peripheral blood cells in children with an increased genetic risk for T1D and at different stages of autoimmunity were investigated in this study. The calculated autoimmunity burden and measured HLA-DQ cell surface MFI in this study provides an indication of a trend of lower HLA-DQ cell surface expression in children at an increased risk for T1D and increasing autoimmunity burden.

## 5 Conclusions

Taken together, our flow cytometric analysis of HLA-DQ cell surface MFI suggest that there is an association of HLA-DQ cell surface expression and increasing number of autoantibodies associated with HLA-DQ2/8 risk. The HLA-DQ antibody, clone REA303, recognizes all different epitopes of HLA-DQ regardless of the HLA background of the subjects. The increased autoimmunity burden, in our study defined as either sAB or cAB, that was related to the decreased HLA-DQ cell surface MFI may be a marker of T cell exhaustion. Further studies of HLA-DQ cell surface expression to better understand the potential role of the autoimmunity burden and HLA in the pathogenesis of type 1 diabetes are therefore warranted.

## Ethics statement

The Diabetes Prediction in Skåne (DiPiS) study was approved by the Regional Ethics Board in Lund (Dnr 2009/244) with the amendments to obtain increased blood volumes (Dnr 2014/196, 2015/861) in order to study children with increased genetic risk for type 1 diabetes, who were followed longitudinally since birth without or with the development of beta cell autoantibodies.

## CRediT authorship contribution statement

**Agnes Andersson Svärd:** Conceptualization, Methodology, Validation, Formal analysis, Investigation, Data curation, Writing - original draft, Writing - review & editing, Visualization, Project administration, Funding acquisition. **Marlena Maziarz:** Formal analysis, Data curation, Writing - original draft, Writing - review & editing, Visualization. **Anita Ramelius:** Methodology, Writing - review & editing. **Markus Lundgren:** Resources, Writing - review & editing. **Åke Lernmark:** Conceptualization, Investigation, Resources, Writing - original draft, Writing - review & editing, Supervision, Funding acquisition. **Helena Elding Larsson:** Conceptualization, Investigation, Resources, Writing - original draft, Writing - review & editing, Supervision, Funding acquisition.

## Declaration of competing interest

None.
