# Supplementary material for: Decreased HLA-DQ expression on peripheral blood cells in children with varying number of beta cell autoantibodies
Source: J Transl Autoimmun. 2020 Apr 9;3:100052. doi: 10.1016/j.jtauto.2020.100052 (PMC7388396; doi:10.1016/j.jtauto.2020.100052)
Supplement: Multimedia component 1 [file mmc1.docx]

**Supplemental Figure 1. Representative flow cytometry data of Kaluza analysis on isolated cell subsets, all from the same subject.** The analyses follow the same format, cells are stained with the appropriate antibody with conjugated fluorescence marker (grey). For each cell subset, purity of cells is presented on the top row and HLA-DQ cell surface fluorescence on the bottom row. Up to 10,000 events are recorded in the initial gate. Unstained PBMC were used as negative control (dotted, red). To identify populations of neutrophils (CD16^+^CD66^+^) and monocytes (CD14^+^CD16^-^), these samples were stained with multiple antibodies and FMOs were used. From the initial gate, 90.3% and 96.5% of events were observed as neutrophil (CD16^+^CD66^+^) and monocyte (CD14^+^CD16^-^) populations, respectively. The analysis is representative of the data presented in Table 3.

**Supplemental Figure 2. Schematic of single autoantibody profiles for the n = 67 children in our study during follow-up as part of the DiPiS (Diabetes Prediction in Skåne) study follow-up.** Autoantibody profiles of GADA (panel A), IA2A (panel B), IAA (panel C) and three variants of ZnT8A against arginine, tryptophan or glutamine at position 325 (R/W/Q, respectively) (panel C). The timeline plot shows the visits (circles for visits as part of DiPiS follow-up, stars for time of sampling into our study) and autoantibody count (0 = green, 1 = yellow, and for ZnT8A in (panel D) 2 = red, 3 = purple).

A: GADA

B: IA2A

C: IAA

D: ZnT8A

**Supplemental Figure 3. Estimates and 95% confidence intervals of the association between HLA-DQ cell surface median fluorescence intensity (MFI) on isolated peripheral blood cells and (A) HLA-DQ2/8, (B) autoimmunity burden measured as the number of autoantibodies detected at the time of sampling (sAB), (C) cumulative (cAB) measured as area under the trajectory of autoantibodies over time.** The models for (A) HLA-DQ2/8 were adjusted for age and sex (Model 1) and complete blood count (CBC) of white blood cells, red blood cells and platelets in addition to the parameters in Model 1 (Model 2). The models were fit using linear regression with model-based standard errors. (See the Supplemental Table 2 panel D-F, respectively, for detailed results corresponding to these plots).

**Supplemental Table 1. Frequency table of HLA-DRB345-DRB1-DQA1-DQB1-DPA1-DPB1 alleles determined by Next Generation Sequencing.** Extended HLA haplotypes were determined from the allelic information, an online database (Allele Frequencies in Worldwide Population, http://www.allelefrequencies.net) and literature.

**Supplemental Table 2. Estimates, 95% confidence intervals and the associated p-values (Est (95% CI), p) of the association between HLA-DQ cell surface median fluorescence intensity (MFI) on isolated peripheral blood cells and HLA-DQ2/8 (panels A and D), autoimmunity burden measured as the number of autoantibodies detected at the time of sampling (sAB) (panels B and E), or the cumulative (cAB) measured as area under the trajectory of autoantibodies over time (panels C and F).** The models for HLA-DQ2/8 were adjusted for age and sex (Model 1) and complete blood count (CBC) of peripheral blood cells, red blood cells and platelets in addition to the parameters in Model 1 (Model 2). The models were fit using linear regression with robust standard errors in panels A-C and linear regression with model-based standard error estimation in panels D-F (See Figure 4 and Supplemental Figure 3 for forest plots corresponding to the Tables A-F). None of the p-values remained significant after adjustment for multiple comparisons (corrected using the Benjamini-Hochberg procedure assuming 420 comparisons and a 5% false discovery).
